# Progressive Ventricles Enlargement and Cerebrospinal Fluid Volume Increases as a Marker of Neurodegeneration in Patients with Spinal Cord Injury: A Longitudinal Magnetic Resonance Imaging Study

**DOI:** 10.1089/neu.2017.5522

**Published:** 2018-12-13

**Authors:** Maryam Seif, Gabriel Ziegler, Patrick Freund

**Affiliations:** ^1^ Spinal Cord Injury Center, University of Zurich, Zurich, Switzerland.; ^2^ Department of Neurophysics, Max Planck Institute for Human Cognitive and Brain Sciences, Leipzig, Germany.; ^3^ Dementia Research, Otto-von-Guericke-University, Magdeburg, Germany.; ^4^ German Center for Neurodegenerative Diseases (DZNE), Magdeburg, Germany.; ^5^ Wellcome Trust Centre for Neuroimaging, UCL Institute of Neurology, UCL, London, United Kingdom.

**Keywords:** brain volume, CSF volume, spinal cord injury, VBM, ventricle enlargement

## Abstract

Next to gray and white matter atrophy, cerebrospinal fluid (CSF) volume and ventricular dilation may be surrogate biomarkers for brain atrophy in spinal cord injury (SCI). We therefore aimed to track brain atrophy by means of CSF volume changes and ventricular enlargements over two years after SCI. Fifteen patients with SCI and 18 healthy controls underwent a series of T1-weighted scans during five time points over two years. Changes of CSF/intracranial volume (CSF/ICV) ratio, CSF volume, and ventricular enlargement rate over time were determined. Sample sizes with 80% power and 5% significance were calculated to detect a range of treatment effects for a two-armed trial. There was a significant cross-sectional increased CSF/ICV ratio in patients compared with controls at each time point (*p* < 0.02). The rate of CSF/ICV changes, however, was not significantly different between groups over time. CSF volume increased linearly over bilateral sensorimotor cortices (left: *p* = 0.002, right: *p* = 0.042) and in the supracerebellar space (*p* < 0.001) within two years. An acceleration of the enlargement within the third (*p* = 0.017) and the fourth (*p* = 0.006) ventricles was observed in patients over time. Sample size estimation for six-month trials with CSF volume requires 25 patients per treatment arm to detect a hypothetical treatment effect in terms of slowing of atrophy rate of 30%. This study shows that SCI-induced changes in CSF/ICV ratio and ventricular expansion rate provide additional information on the neurodegenerative processes after injury. The sensitivity to scoring treatment effects speaks to its potential to serve as a sensitive biomarker in addition to local atrophy measures.

## Introduction

Traumatic spinal cord injury (SCI) leads in most patients to profound neurological dysfunction and paralysis below the level of lesion^1^ as information flow between supraspinal and spinal neuronal networks is impaired.^2^ Functional recovery after human SCI is restricted but can be fostered by intensive neurorehabilitation. The neuronal mechanisms underlying neurological and functional recovery, however, are still not well understood because of the complex relationship between neurodegeneration and plasticity. The ability to track trauma-induced structural changes across the neuraxis provides the opportunity to quantify neurodegeneration *in vivo*^3^ and recovery-related plasticity,^4^ which may identify new treatment targets.

After SCI, serial magnetic resonance imaging (MRI) studies revealed progressive neurodegeneration, both in the gray matter (GM) and white matter (WM), along the entire trajectory of the motor^5^ and sensory systems^6^ above the level of injury. Recently, a follow-up study in the same patient cohort revealed that these changes continued for at least two years post-SCI.^7^ Crucially, the magnitude of neurodegeneration at the level of the spinal cord, brainstem, and cortex over the first six months predicted clinical outcome at two years, independent of early clinical changes.^7^

Nevertheless, potential neuroimaging biomarkers for global brain atrophy such as intracranial volume (ICV), cerebrospinal fluid (CSF) volume changes, and ventricular enlargement were not investigated. ICV, CSF volume, and ventricle enlargement measurements are reliable morphometric features to determine atrophy patterns in patients with mild cognitive impairment, Alzheimer disease,^8^ Parkinson disease,^9^ Huntington disease,^10^ and traumatic brain injury (TBI).^11^ For instance, early ventricular dilatation was observed in the course of significant cognitive decline in patients with Parkinson disease.^9^ In addition, it has been shown that the CSF/ICV ratio, besides its potential to quantitate general brain atrophy,^12^ does not depend on sex and therefore may be used in mixed sex studies as well.^13^

The rationale for the use of these markers of global brain atrophy measures in SCI arises as the trauma to the spinal cord triggers a cascade of inflammatory processes that spreads across the central nervous system (CNS).^14–17^ Chronic inflammation is associated with neurodegeneration and hence could lead to volumetric fluctuations in brain tissue.^18,19^ Interestingly, measures of global brain atrophy as well as longitudinal MRI findings are now used as surrogate end-points in clinical trials^9,10,19^ next to measures of focal GM and WM neurodegeneration to complement clinical assessments for disease-modifying trials. It remains to be established, however, whether MRI-derived measurement of global brain atrophy and ventricle expansions (both are reflected by increases in CSF volume) are sensitive and accurate in identifying disease-related changes in patients with SCI.

The aim of this study was to investigate the trajectory of progressive global brain atrophy (i.e., CSF/ICV ratios, volumetric CSF changes) and local brain atrophy (i.e., ventricular enlargement) over two years in the same SCI patient cohort that previously showed enduring neurodegenerative changes in the cortical GM and WM after SCI.^7^ To track brain atrophy, we applied longitudinal Voxel Based Morphometry (VBM)^20,21^ on serially acquired high resolution T1-weighted MR images. We estimated the sample sizes for a six-month trial of CSF volume that might inform the design of future clinical trials to detect a range of treatment effects with 80% statistical power.

## Methods

### Subjects

The longitudinal study was approved by the local ethics committee of Zurich (EK-2010-0271), and written informed consent was obtained from each subject before the examination. Fifteen patients with SCI (nine tetraplegic and six paraplegic patients, mean age, 48 years ±19; age range, 19–75 years, Table 1) and 18 healthy controls (mean age, 35 years ±10, age range, 23–65 years) underwent a series of T1-weighted three-dimensional Magnetization Prepared Rapid Acquisition Gradient Echo, (3D-MPRAGE) scans during five time points over two years. The inclusion criteria were traumatic subacute SCI patients with no head and brain lesions, no mental or medical disorders affecting functional results. Patients underwent a comprehensive clinical assessment, including the International Standards for the Neurological Classification of Spinal Cord Injury protocol^22^ at baseline and at two months, six months, 12 months, and 24 months follow-up.

**Table 1.** Clinical Data of 15 Patients with Subacute Traumatic Spinal Cord Injury

*ID*	*Age (years)*	*Severity of injury*	*AIS grade at baseline*	*Initial level of impairment (motor/sensory)*
1	19	Complete	A	C5/C4
2	23	Incomplete	B	C7/C6
3	70	Incomplete	B	T10/T10
4	75	Incomplete	D	T12/T12
5	44	Incomplete	D	T11/T11
6	42	Complete	A	C5/C5
7	71	Incomplete	B	C7/C8
8	20	Complete	A	C5/C5
9	30	Incomplete	B	C7/C8
10	52	Incomplete	D	T9/T9
11	42	Incomplete	D	C5/C4
12	29	Complete	A	T11/T11
13	70	Complete	A	T7/T7
14	52	Incomplete	B	C6/C6
15	68	Incomplete	D	C4/C4

### MRI measurements

The 3D-MPRAGE sequence comprises the following parameters: field of view = 224 × 256 mm^2^, matrix size = 224 × 256, repetition time = 2420 msec, echo time = 4.18 msec, readout bandwidth = 150 Hz per pixel, 1 mm^3^ of resolution, flip angle α = 9 degrees, inversion time = 960 msec, and total acquisition time of 9 min. The first scan (baseline) was acquired at 49.67 (± 22) days post-injury, the second scan at two, the third scan at six, the fourth scan at 12, and the fifth scan at 24 months after injury. The images on the first four time points were acquired using a 3T Magnetom Verio (Siemens Healthcare, Erlangen, Germany), and for measurements on the fifth time point, the scanner was upgraded to a 3T Magnetom Skyra^fit^. We used a 16-channel radiofrequency receive head and neck coil in combination with a spine matrix coil.

Trained radiographers positioned all participants in the same supine position for each scan. Image acquisition over five time points was completed successfully in 14 patients and in 18 healthy controls. One patient died of causes unrelated to SCI after the second time point. A total of 156 MRI datasets were analyzed from 33 participants. All T1-weighted 3D-MPARGE images acquired from subjects were included in the VBM analysis.^20,21^

### Brain volume

Global tissue volumes for GM, WM, and CSF at each time point were calculated using the segmented T1-weighted images with applying unified segmentation,^23^ and the total ICV was expressed as the sum of volumes of all tissue classes. A previously established global measure of CSF volume is the CSF volume-to-ICV (CSF/ICV) ratio, calculated as the CSF volume divided by total brain volume (sum of GM, WM, and CSF) to adjust for intersubject differences in brain size.^24^ CSF/ICV ratio is used as a global atrophy marker associated with CSF volume.

To assess local change of CSF volume over time, longitudinal VBM was applied within SPM12 (Wellcome Trust Centre for Neuroimaging). Diffeomorphic registration was applied for longitudinal MRI, and resulting midpoint images were segmented,^20^ Non-linear template generation and image normalization were performed using a geodesic shooting procedure.^25^ The template was affine registered to the standard brain template from Montreal Neurological Institute for all subsequent modeling steps. Consecutively, normalized CSF tissue segments from all subjects and time points were modulated by the Jacobian determinants encoding individual volume changes over time. Morphometric images were smoothed using Gaussian kernels of 6 mm full width at half maximum. Subsequent modeling and analysis were performed for smoothed, normalized CSF segments within specific brain areas.

### Statistical analysis

To statistically assess cross-sectional and longitudinal changes of the CSF/ICV ratio, we used pairwise comparisons for each time point and linear mixed effects models with a group indicator and group by time interaction to assess changes over time using STATA 14 (Stata Corp LP, College Station, TX).

To assess group differences in trajectories of local CSF volume and ventricular enlargements, we followed a conservative two-stage summary statistics approach commonly used in fMRI and longitudinal image analysis. In a first stage, we estimated individual quadratic trajectory models y (t) = β_0_ + β_1_ t + β_2_ t^2^ and obtained intercepts (β_0_), rate of change (β_1_), quadratic effects (β_2_), and time since injury (t) for all subjects in the sample independently. In a second stage, we used two-sample parametric *t* tests (for all voxels within each region of interest [ROI]), comparing the parameters across clinical groups, while adjusting for age and sex as covariates of no interest. Group differences of linear (e.g., β_1_ < 0 indicating decline) and quadratic (e.g., β_2_ > 0 indicating deceleration) effects were assessed using random field theory for correction of multiple comparisons within each considered ROI.

The associated *p* values were corrected for multiple comparisons using family-wise error correction, and cluster significance was tested (after applying a cluster-forming–threshold of 0.001), using Gaussian random field theory. Regression models were applied to determine associations between CSF volume and ventricular expansion and clinical outcomes over two years of follow-up. The mean age difference between patients and controls was not found to be statistically significant (*p* = 0.071, Mann–Whitney *U* test). However, age was included as a covariate of no interest in all statistical tests.

For CSF volume, we applied the six-month effect size to calculate estimates of the sample sizes necessary to detect a 100% treatment effect with 80% statistical power and 5% significant differences between healthy controls and patient group, by use of the standard formula based on two-group trials, assuming a baseline adjusted comparison of mean (analysis of covariance).^26^ The required Pearson correlation coefficient between baseline data and six-month CSF volume was estimated using the available data. The associations between MRI readouts (i.e., CSF/ICV ratio and ventricle enlargements) and clinical outcomes were investigated using regression model in SPM 12 and Stata 13.

## Results

The local WM and GM changes over two years were estimated previously from these 33 participants, and the results have been presented before.^7^ The mean scan intervals between time of injury and imaging baseline, two, six, 12, and 24 months were 49.67 (standard error of the mean [SE] 5.91), 103.5 (SE 12.40), 220.36 (SE 18.69), 389.93 (SE 29.60), and 881.14 (SE 43.07) days, respectively.

### Global CSF volume

**FIG. 1.**
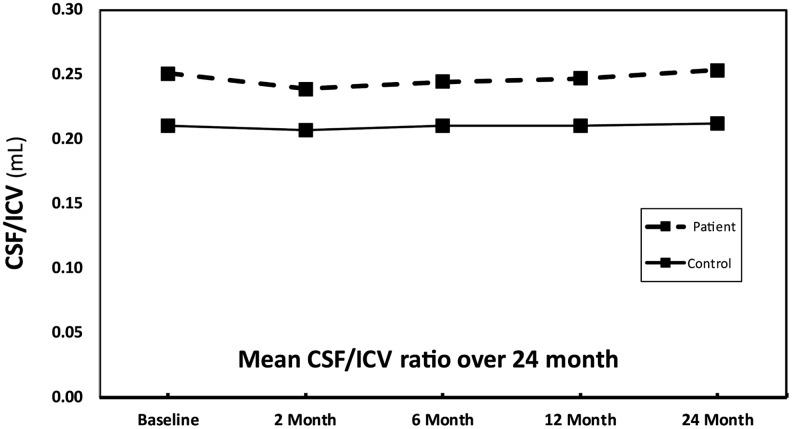
The cerebrospinal fluid/intracranial volume (CSF/ICV) ratio across all time points in patients with spinal cord injury and controls. The CSF/ICV ratio significantly increased at baseline and remained increased over time in patients compared with controls accounting for covariates. There was no significant difference within rates of CSF/ICV ratio at different time points.

In this study, at baseline, CSF/ICV ratio was significantly increased in patients compared with controls (patients = 0.21 ± 0.03 mL; controls = 0.25 ± 0.05 mL, *p* < 0.01). The ratio remained constantly increased (i.e., cross-sectional), but the linear slope of rate of change was not significantly different between patients and controls over time (Fig. 1).

### Local CSF volume and ventricles enlargement

**FIG. 2.**
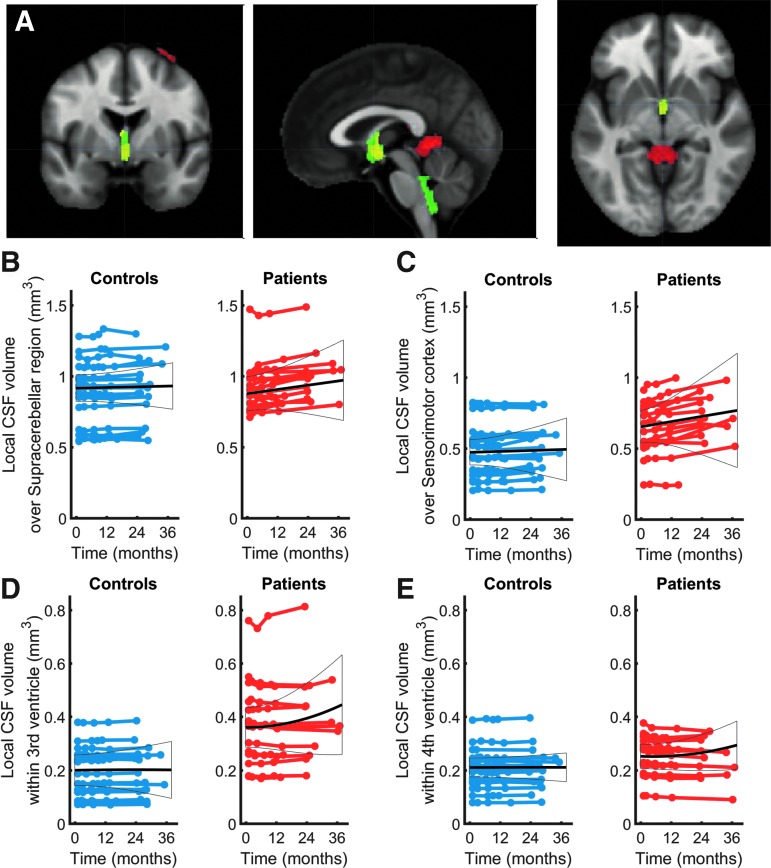
Longitudinal changes in local cerebrospinal fluid (CSF) volume and ventricle enlargements, shown by longitudinal voxel based morphometry (VBM) model. (**A**) Overlay of statistical parametrical maps showing regions of volume changes in local CSF volume: yellow is baseline intercept, red is linear term, and green is quadratic. (**B,C,D,E**) Individual longitudinal data points for controls (blue) and patients (red); colored lines connect the data from each individual. Black solid lines depict the fitted group model describing average progression in the whole group. Linear (B,C) and quadratic (D,E) group progression models were fitted. All models explicitly account for effects of covariates of no interest such as age. Color image is available online at www.liebertpub.com/neu

At baseline, the third ventricle was enlarged (z score = 3.81, *p* = 0.011) in patients relative to controls while accounting for age and sex. Over 24 months, local CSF volume increased linearly (i.e., degeneration) over bilateral sensorimotor cortices (left: z score = 4.14 *p* = 0.002, right: z score = 4.21, *p* = 0.042) and within the left supracerebellar space (z score = 4.97, *p* < 0.001, Fig. 2A,B,C and Table 2). Testing for potential effects of recovery in terms of deceleration and acceleration of the disease process, we found that the third (z score = 4.60, *p* = 0.017) and fourth (z score = 3.82, *p* = 0.006) ventricle enlargements accelerated (positive quadratic effect) in patients compared with the controls (Fig. 2A,D,E) over time.

**Table 2.** Cerebrospinal Fluid Volume and Ventricle Size Changes in Patients with Spinal Cord Injury

	*Z-Score*	*P* *(FWE-corrected)*	*Cluster extent*	*X* *(mm)*	*Y* *(mm)*	*Z* *(mm)*
Baseline local CSF volume increase						
	3.81	0.011	190	−3	−3	−8
Rate of local CSF volume and ventricle size change						
CSF (left M1/S1)	4.14	0.002	388	−21	−39	80
CSF (right M1/S1)	4.21	0.042	209	33	−8	71
CSF(left supracerebellar space)	4.97	<0.001	1340	−3	−50	2
Non-linear group differences in ventricle size						
Deceleration of third ventricle expansion	4.60	0.017	305	0	−3	6
Deceleration of fourth ventricle expansion	3.82	0.006	380	−5	−41	−29
^*^ Only clusters with significant rates of change between patients and controls are shown.						

CSF, cerebrospinal fluid.

### Clinical outcomes correlation

The associations between MRI readouts (i.e., CSF/ICV ratio and ventricle enlargements) and clinical outcomes showed no significant correlations between MRI parameters and clinical outcome measures.

### Sample sizes

**FIG. 3.**
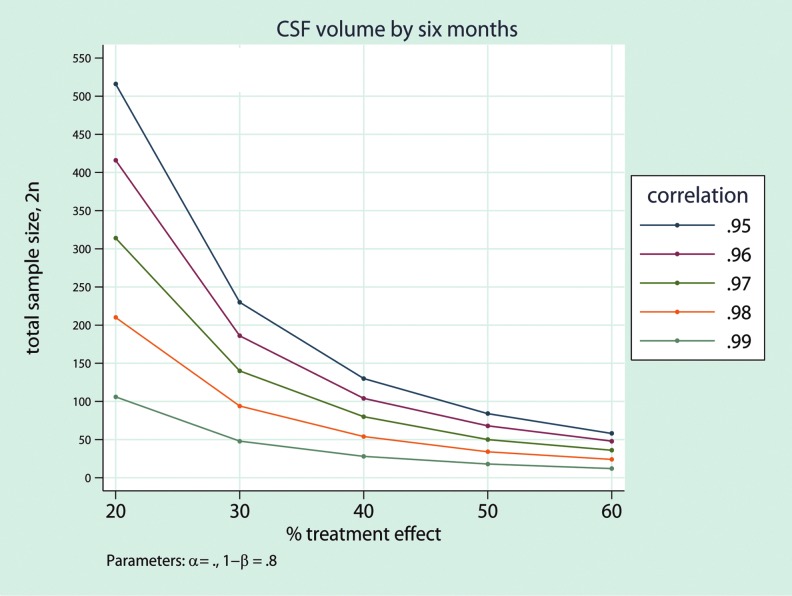
The relation between the required sample size for a six-month randomized clinical trial with 80% statistical power and effect size for a range of potential treatment efficacies shown for cerebrospinal fluid (CSF) volume. The calculations cover a range of plausible baseline versus six-month correlations around the observed patient values of 0.99. Color image is available online at www.liebertpub.com/neu

Figure 3 demonstrates the sample size requirements for six-month clinical trials that have 80% statistical power at 5% significance to detect a 30% treatment effect. For instance, in a six-month trial that has 80% statistical power (at a two-sided 5% significance level) to detect 30% changes in CSF volume using VBM model, 25 patients are required per group.

## Discussion

This longitudinal study shows that progressive global brain atrophy is evident—next to GM and WM atrophy as reported in the same cohort—early after SCI. At baseline, the CSF/ICV ratio had increased already and remained so over time without substantial fluctuations while changes of CSF volume over the sensorimotor cortex and within the supracerebellar space showed sustained increases. Interestingly, the rate of expansions of the third and fourth ventricle showed linear and non-linear changes (i.e., acceleration of the disease process) over time. These dynamic volumetric expansions may be indicative of enduring neurodegenerative processes within the cortical GM and WM as reported in this cohort before over two years post-SCI.^7^ Sample size calculations demonstrated the sensitivity of acute CSF neuroimaging biomarkers to render them viable candidates for scoring the effects of treatment, including rehabilitation.

Several studies^5–7^ focused previously on progressive GM and WM atrophy after SCI but did not address global measures of brain atrophy such as the CSF/ICV ratio, local CSF volume, and ventricular expansion. Although non-specific to any single disease process, global measures of brain atrophy have proven sensitive in tracking disease processes and in picking up treatment-induced changes in neurodegenerative diseases,^8–10^ thus rendering them viable tools in clinical trials.^10^ Moreover, a previous MRI study on 123 patients with TBI showed that TBI results in the expansion of CSF spaces, particularly in the temporal horns and third ventricle, which preceded subsequent reduction in total brain volume.^11^ A gradual process of diminishing arborisation of surviving neurons as a result of disruption of neuronal circuitry might be one possible explanation for this observation.^11^

In the current SCI cohort, next to baseline differences of the CSF/ICV ratio and enlarged third and fourth ventricle, linear increases of CSF volume were detectable in the supracerebellar space and bilaterally over the sensorimotor cortices. The increase of CSF volume over the sensorimotor cortices might reflect active neurodegeneration within the GM in the output deprived leg representing area of primary motor cortex.^7^ The reported focal GM changes within the sensorimotor cortices (restricted to the leg area) and thus at the border to the CSF^27^ might be because of increases in CSF volume, however, which had been assigned falsely to GM during segmentation.

Cognitive decline, anxiety, and depression are reported to be elevated in patients with SCI when compared with the normal population.^28^ Crucially, patients with depression showed a higher CSF volume in other neurodegenerative diseases,^29^ which may also be the case in SCI.^16^ Because these symptoms have been associated with brain atrophy in other diseases,^29^ it seems likely that global brain atrophy in our cohort could be associated with signs of cognitive impairment. In particular, we have shown previously that the limbic system in this cohort shows signs of neurodegeneration.^5,6^

We did not anticipate that the global measures of the CSF changes were related to specific functional measures of recovery. This finding is in line with a previous study in Huntington disease in which no significant correlations were detected between ventricular volume changes and clinical measures.^10^ Changes in imaging outcomes in response to treatment might not always be accompanied by functional improvements, and clinical function might sometimes improve in the absence of changes in imaging measures.^10^

Thus, although anticipated, our results provide no evidence that the severity of trauma is related to the rate of change of CSF measures. This is interesting because it also points to the fact that a focal CNS injury, such as SCI, might induce a cascade of secondary neurodegenerative events that progress with a distinct time profile. The conjoint analysis of CSF volume has the potential to elucidate whether the chronic inflammatory process within the CSF or the WM and GM is the main factor driving the observed changes in our study cohort.

Based on the estimated longitudinal effect sizes of CSF volume, we recommend CSF volume as an outcome measures to power clinical trials in SCI. These MRI-based measures may afford the opportunity to assess site-specific effects of intervention, essential for the translation of trial efficacy into clinical effectiveness.^30^ Hypothetical treatment effects, defined by slower longitudinal structural changes in these imaging measures, could be detectable over a realistic time scale with significantly lower sample sizes than required for traditional clinical readouts.^31^ Thus, these objective outcome measures hold considerable promise for quantifying the effects of treatment. In short, quantitative MRI biomarkers of neurodegeneration therefore represent promising instruments for the stratification of patient cohorts and the improvement of trial efficiency.^30^

### Limitations

A limitation of the study is the relatively small number of participants that were recruited. In total, however, 156 data points from patients and controls were included in the analysis and the summary statistics used maximize the efficiency because all data points were included. Hypothetical treatment effects defined by slower longitudinal structural changes in these measures would be detectable over a realistic time scale with practical sample sizes and be useful in monitoring trauma-induced changes before, during, and after treatment. Our sample size calculations are made under several assumptions. We do consider full compliance, no dropout, any possible effects of treatment response heterogeneity, all of which might inflate within group variance. The impact of these confounds, however, is an order of magnitude smaller than the sample sizes that we present.

## Conclusion

We found that CSF/ICV ratio, CSF volume, and ventricular enlargement rate are sensitive to neurodegenerative changes in SCI by way of group differences between patients and healthy controls. The sensitivity to scoring treatment effects speaks to its potential to serve as a sensitive biomarker in addition to local GM and WM atrophy measures. The link between inflammatory effects detectable within the CSF^32,33^ and global brain atrophy should be addressed next.
